# A Novel MgO-CaO-SiO_2_ System for Fabricating Bone Scaffolds with Improved Overall Performance

**DOI:** 10.3390/ma9040287

**Published:** 2016-04-14

**Authors:** Hang Sun, Shiwei He, Ping Wu, Chengde Gao, Pei Feng, Tao Xiao, Youwen Deng, Cijun Shuai

**Affiliations:** 1 State Key Laboratory of High Performance Complex Manufacturing, Central South University, Changsha 410083, China; shsunhang@csu.edu.cn (H.S.); gaochengde@csu.edu.cn (C.G.); fengpei@csu.edu.cn (P.F.); 2 School of Basic Medical Science, Central South University, Changsha 410078, China; wishter_he@csu.edu.cn; 3 College of Chemistry, Xiangtan University, Xiangtan 411105, China; pingwu@xtu.edu.cn; 4 Department of Orthopedics, The Second Xiangya Hospital, Central South University, Changsha 410011, China; xiaotaoxyl@163.com (T.X.); drywdeng@163.com (Y.D.)

**Keywords:** MgO-CaO-SiO_2_ system, forsterite, wollastonite, scaffold, overall performance

## Abstract

Although forsterite (Mg_2_SiO_4_) possesses good biocompatibility and suitable mechanical properties, the insufficient bioactivity and degradability hinders its further application. In this study, a novel MgO-CaO-SiO_2_ system was developed by adding wollastonite (CaSiO_3_) into Mg_2_SiO_4_ to fabricate bone scaffolds via selective laser sintering (SLS). The apatite-forming ability and degradability of the scaffolds were enhanced because the degradation of CaSiO_3_ could form silanol groups, which could offer nucleation sites for apatite. Meanwhile, the mechanical properties of the scaffolds grew with increasing CaSiO_3_ to 20 wt %. It was explained that the liquid phase of CaSiO_3_ promoted the densification during sintering due to its low melting point. With the further increase in CaSiO_3_, the mechanical properties decreased due to the formation of the continuous filling phase. Furthermore, the scaffolds possessed a well-interconnected porous structure and exhibited an ability to support cell adhesion and proliferation.

## 1. Introduction

For bone scaffolds, the excellent bioactivity, controlled degradability, osteo-conductive and appropriate mechanical properties were indispensable in meeting clinical requirements [1,2,3,4]. Forsterite (Mg_2_SiO_4_), the MgO-SiO_2_ system bioceramic, possessed good cytocompatibility and suitable mechanical properties [5,6]. Magnesium, as an important mineral element in bone tissues, played a key role in bone remodeling and skeletal development [7,8]. Silicon, as an indispensable element in human nutrition, was associated with improving bone calcification [9,10], while the low degradability and poor bioactivity hindered its further application in bone repair [11].

Wollastonite (CaSiO_3_), the CaO-SiO_2_ system bioceramic, was a representative bioactive material for tissue regeneration due to its strong ability to induce bone-like apatite formation and a fast degradation rate [12,13,14]. Moreover, it could be used as a reinforcement phase to fill ceramics for further densification [15,16]. Sainz *et al*. developed CaSiO_3_-CaMg(SiO_3_)_2_ eutectic bioceramics to be used as bioactive implant materials, and found that they showed high reactivity and proper degradability in simulated body fluid (SBF) [17]. Chang *et al*. developed hydroxyapatite/wollastonite composite bioceramic for hard tissue repair, suggesting that the introduction of CaSiO_3_ into hydroxyapatite was an effective method to obtain composites with enhanced mechanical properties, proper dissolution rate and improved bioactivity [18].

In this study, CaSiO_3_ was incorporated into Mg_2_SiO_4_ to develop a novel MgO-CaO-SiO_2_ system for fabricating bone scaffolds via selective laser sintering (SLS). The apatite-formation ability, degradability, mechanical properties and the MG63 cell responses to scaffolds were investigated.

## 2. Results and Discussion

### 2.1. Mixed Powders

**Figure 1:**
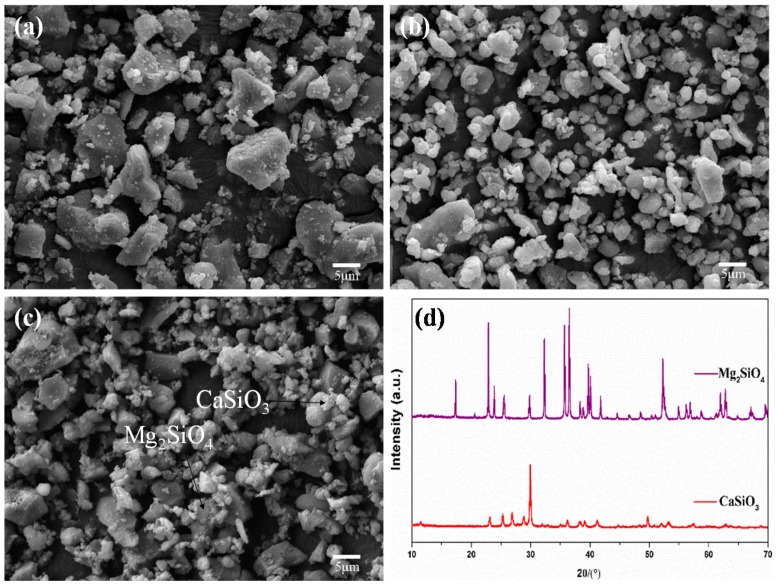
The microstructure of powders: (**a**) initial forsterite (Mg_2_SiO_4_); (**b**) initial wollastonite (CaSiO_3_); (**c**) mixed powders; and (**d**) X-ray diffraction (XRD) patterns.

The Mg_2_SiO_4_ and CaSiO_3_ powders were irregular bulk and approximate sphere, respectively (Figure 1a,b). The small CaSiO_3_ particles (0.2–2 μm) were randomly distributed on and between the Mg_2_SiO_4_ particles (about 5 μm) after mixing (Figure 1c). It might benefit the sintering process due to the large specific surface area of CaSiO_3_. The X-ray diffraction (XRD) patterns of the received Mg_2_SiO_4_ and CaSiO_3_ powders (Figure 1d) were consistent with JCPDS (Joint Committee on Powder Diffraction Standards) cards for Mg_2_SiO_4_ and β-CaSiO_3_ (JCPDS cards No. 34-0189 and No. 84-0654) without the peaks of other phases detected.

### 2.2. Scaffolds

**Figure 2:**
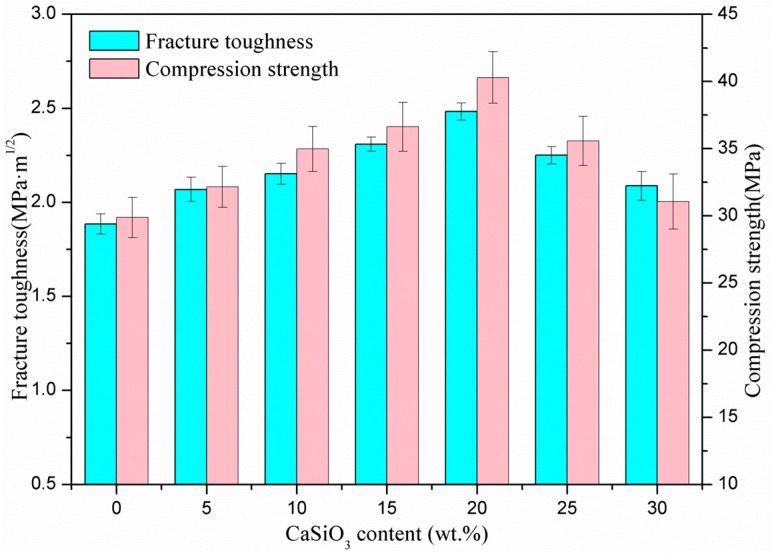
Fracture toughness and compression strength of the scaffolds with different CaSiO_3_ content.

The fracture toughness and compression strength of the scaffolds increased with increasing CaSiO_3_ from 0 to 20 wt %, but began to decrease with further increasing CaSiO_3_ (Figure 2). In this study, when 20 wt % CaSiO_3_ was introduced, the scaffolds obtained an optimal fracture toughness and compressive strength of 2.48 ± 0.05 MPa·m^1/2^ and 40.29 ± 1.32 MPa, which represented an improvement of 31.7% and 34.90% compared to the scaffolds without CaSiO_3_, respectively. It indicated that the mechanical properties of the scaffolds could be effectively strengthened by incorporating CaSiO_3_.

**Figure 3:**
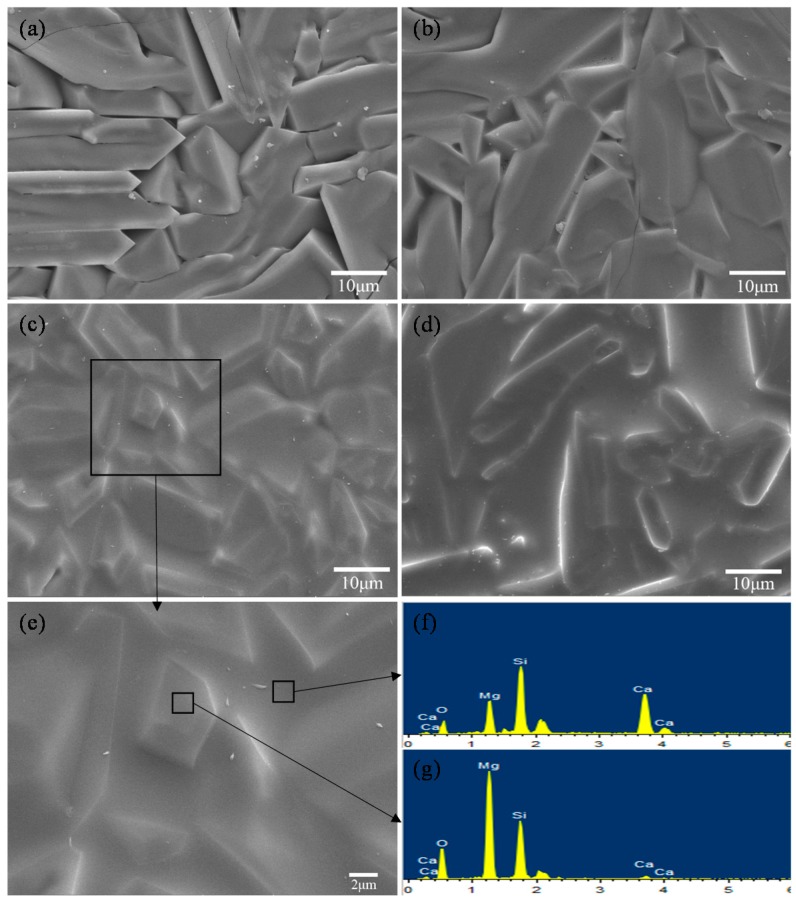
Morphologies of the scaffolds with different CaSiO_3_ content: (**a**) 0 wt %; (**b**) 10 wt %; (**c**,**e**) 20 wt %; (**d**) 25 wt % and (**f**,**g**) energy dispersive spectroscopy spectrums of the scaffold with 20 wt % CaSiO_3_.

The microstructures of the scaffolds are shown in Figure 3. The scaffolds without CaSiO_3_ exhibited distinct Mg_2_SiO_4_ particle appearance with clear boundaries and some clearances between Mg_2_SiO_4_ particles (Figure 3a). In the case of scaffolds with 10 wt % CaSiO_3_, a small amount of liquid phase enhanced the densification by filling the clearances (Figure 3b). For the scaffolds with 20 wt % CaSiO_3_, sufficient liquid phase was formed, which remarkably improved the densification without clearances (Figure 3c,e). The liquid phase was believed to play an important role in improving the mechanical strength of the scaffolds [15,16]. However, when CaSiO_3_ increased to 25 wt % (Figure 3d), the excessive liquid phase formed a continuous filling phase, which resulted in the decline of mechanical properties.

**Figure 4:**
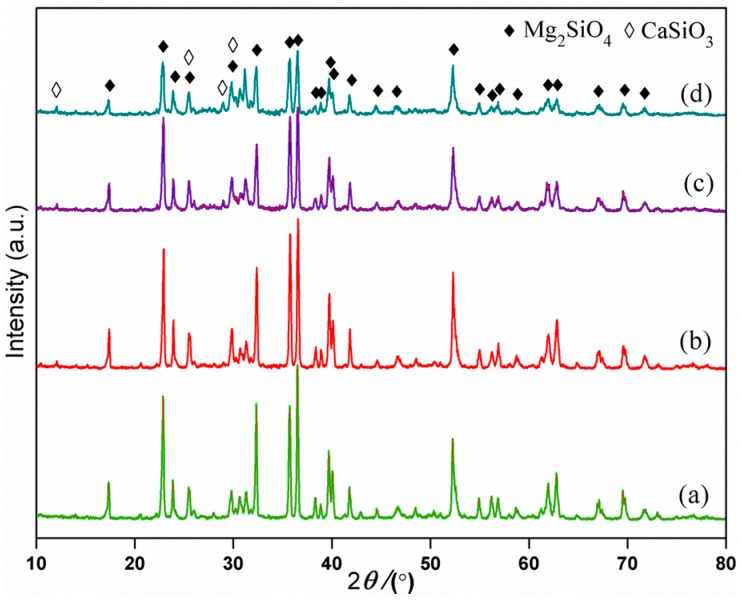
X-ray diffraction patterns of the scaffolds with: (**a**) 0 wt %; (**b**) 10 wt %; (**c**) 20 wt %; and (**d**) 25 wt % CaSiO_3_.

The major diffraction peaks of the scaffolds were consistent with Mg_2_SiO_4_ (JCPDS card No. 34-0189) (Figure 4), which meant that the scaffolds were mainly composed of Mg_2_SiO_4_. For the scaffolds with 10, 20, and 25 wt % CaSiO_3_, some peaks of β-CaSiO_3_ (JCPDS card No. 84-0654) at 2θ = 11.48°, 25.20°, 28.80°, and 30.00° were detected, and gradually increased in intensity with the increase of CaSiO_3_ (Figure 4b–d). Meanwhile, no distinct peaks of other phase were detected.

**Figure 5:**
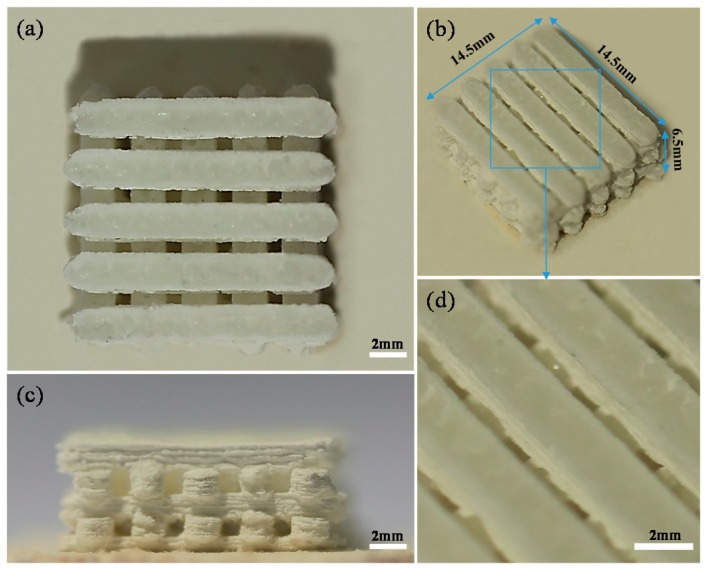
The scaffold with 20 wt % CaSiO_3_: (**a**) top view; (**b**) isometric view; (**c**) lateral view; and (**d**) enlarged partial view.

A scaffold with 20 wt % CaSiO_3_ was fabricated with geometric dimensions of 14.5 mm × 14.5 mm × 6.5 mm (Figure 5). It possessed an interconnected porous structure with a pore size of about 500–800 μm. Such porous microstructure was beneficial for cell ingrowth, nutrients transport and waste products excreted from the scaffold. These demonstrated that SLS have a good ability to fabricate interconnected porous structure of the scaffolds.

### 2.3. Degradability

**Figure 6:**
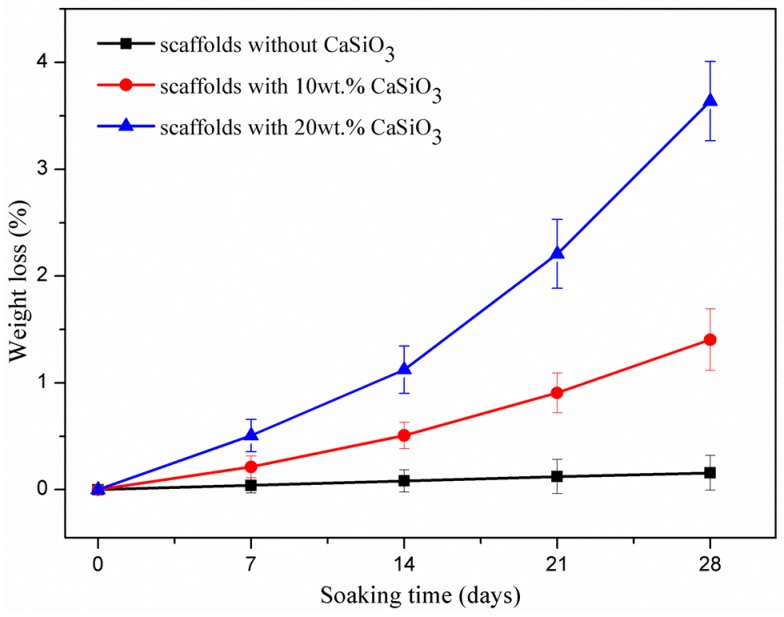
Weight loss of the scaffolds in phosphate buffer saline (PBS) solution.

Degradability is an important requirement for the bone scaffold to match the process of tissue repair or regeneration [19]. The scaffolds without CaSiO_3_ hardly degraded; on the contrary, the scaffolds with CaSiO_3_ continuously degraded after immersion, and the degradation rate increased with the increase of the CaSiO_3_ (Figure 6). The weight loss percentage of the scaffolds with 20 wt % CaSiO_3_ was 0.51% on day 7. With the prolonging of the immersion time, it remarkably increased to 3.64% on day 28. As expected, the scaffolds exhibited an improved degradability when CaSiO_3_ was introduced. The differences of dissolution behavior between Mg_2_SiO_4_ and CaSiO_3_ were owing to their difference in chemical composition and crystalline structure [20].

### 2.4. Bioactivity

**Figure 7:**
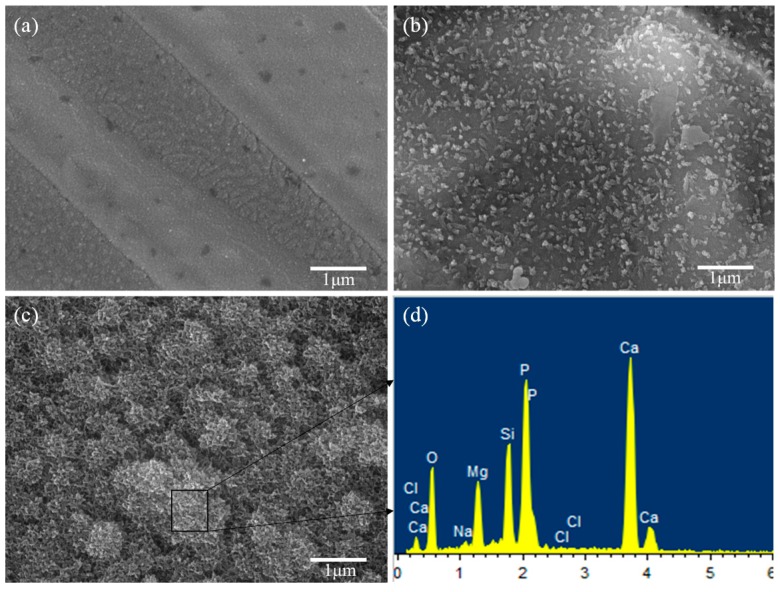
Morphologies of the scaffolds with: (**a**) 0 wt %; (**b**) 10 wt %; (**c**) 20 wt % CaSiO_3_ after immersion in simulated body fluid (SBF) for 14 days; and (**d**) EDS spectra at the location indicated with the square.

The scaffolds without CaSiO_3_ showed a smooth surface after soaking in SBF for 14 days (Figure 7a), which meant that the Mg_2_SiO_4_ was short of apatite formability. For the scaffolds with 10 wt % CaSiO_3_ (Figure 7b), some worm-like particles appeared and uniformly distributed on the scaffolds’ surface after 14 days of soaking. With the CaSiO_3_ increasing to 20 wt % (Figure 7c), the scaffold surface was covered by a dense layer of cauliflower-like precipitates. Furthermore, the elements Ca and P, the main constituent elements of apatite, were detected from an energy dispersive spectroscopy analysis of the cauliflower-like precipitates, which indicated that these precipitates were the bone-like apatite.

**Figure 8:**
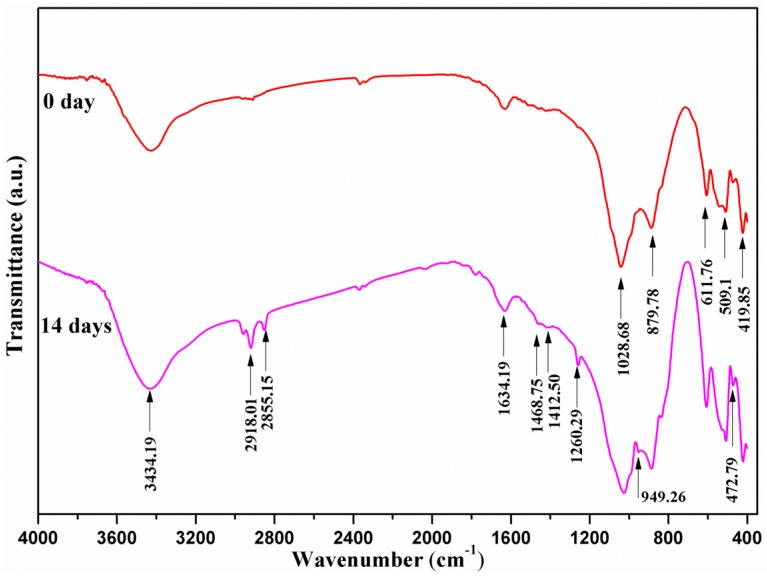
Fourier transform infrared spectrums of the scaffolds with 20 wt % CaSiO_3_ before and after soaking in SBF.

Fourier transform infrared (FTIR) spectra of the scaffold with 20 wt % CaSiO_3_ before and after 14 days of soaking in SBF were recorded, ranging from 400 to 4000 cm^−1^ (Figure 8). Before soaking (day 0), absorption bands related to Mg_2_SiO_4_ and CaSiO_3_ were detected at the wavenumber of 611.76 and 509.10 cm^−1^ for SiO_4_ bending, 1028.68 and 879.78 cm^−1^ for SiO_4_ stretching, and 472.79 and 419.85 cm^−1^ for octahedral MgO_6_ [21,22]. Additionally, the bands at 3435 and 1634 cm^−1^ belonged to the O–H bond of the hydroxyl groups [22]. After 14 days of soaking, new bands at 2918.01 and 2855.15 cm^−1^ were assigned to the O–H group in the hydroxyapatite [23]. The bands observed at 1260.29 and 949.26 cm^−1^ belonged to the P–O bending, which is also similar to hydroxyapatite [24,25]. Those bands at 1412.50 and 1468.75 cm^−1^ were in accordance with the bands of carbonate groups in hydroxyapatite [26]. The FTIR spectra further demonstrated that bone-like apatite formed on the scaffold with 20 wt % CaSiO_3_ after 14 days of soaking in SBF.

**Figure 9:**
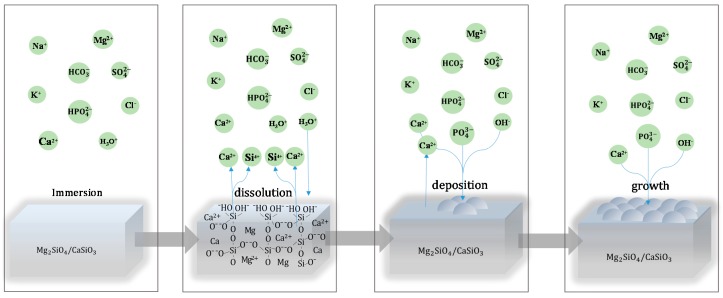
Mechanism of bone-like aspatite formation on the scaffolds with CaSiO_3_ in SBF.

The results indicated that the scaffolds with 20 wt % CaSiO_3_ obtained an improved degradability and apatite-formation ability compared to scaffolds without CaSiO_3_. The mainly reason was that the solubility product constants (*K*_SP_) of CaSiO_3_ (2.5 × 10^−8^) are much bigger than that of Mg_2_SiO_4_ (2.2 × 10^−17^), because the Mg–O bond energy of Mg_2_SiO_4_ was higher than the Ca–O bond of CaSiO_3_ [20,27]. Moreover, CaSiO_3_ could rapidly degrade in SBF and form a silicon-rich layer on the surface, which could induce apatite formation [7]. This apatite formation mechanism in SBF could be summed up as follows (Figure 9) [7,28].

CaSiO_3_ dissolved in SBF and initially released calcium ions (Ca^2+^); subsequently, the Ca^2+^ exchanged with the hydrogen ions (H_3_O^+^) of SBF to form silanol (Si–OH) groups and hydroxyl ion (OH^−^) on the scaffold surface (Equation (1)):

(1) $${\text{CaSiO}}_{3}\left(s\right)+H_{2}O\to {\text{Ca}}^{2+}\left(\text{aq}\right)+{\text{HSiO}}_{3}^{-}\left(\text{aq}\right)+{\text{OH}}^{-}\left(\text{aq}\right)$$

With the degradation of CaSiO_3_, the released Ca^2+^ continually exchanged with H_3_O^+^. As a result, lots of Si–OH groups formed on scaffold surfaces, which were reported to be favorable sites for apatite nucleation and crystallization. Meanwhile, the Ca^2+^ and OH^−^ ions on the dissolved surface continually increased until they exceeded the solubility of the apatite in SBF. Both factors enhanced the driving force to induce the deposition of hydroxyapatite (Equation (2)):

(2) $$10{\text{Ca}}^{2+}\left(\text{aq}\right)+6{\text{HPO}}_{4}^{2-}\left(\text{aq}\right)+8{\text{OH}}^{-}\left(\text{aq}\right)\to {\text{Ca}}_{10}{\left({\text{PO}}_{4}\right)}_{6}{\left(\text{OH}\right)}_{2}\left(s\right)+6H_{2}O$$

The hydroxyapatite crystallized from the amorphous phase to more stable phases. Then, the hydroxyapatite layer could spontaneously grow on the scaffold surface due to the reaction of calcium, phosphate and hydroxyl ions. Additionally, some carbonate-substituted hydroxyapatite formed due to the substitution of ${\text{PO}}_{4}^{3-}$ or OH^−^ ions with the ${\text{CO}}_{3}^{2-}$ ions [28]. The hydroxyapatite was reported to provide a short period of biological bonds with living bone tissue, and it could forecast the *in vivo* bone bioactivity of the scaffolds.

### 2.5. Cell Behavior

**Figure 10:**
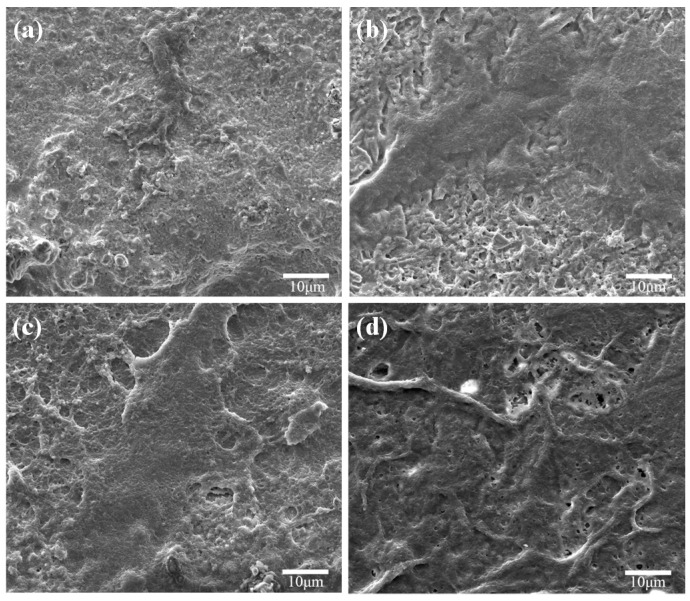
Morphologies of MG63 cells cultured on scaffolds without CaSiO_3_ (**a**,**b**) and scaffolds with 20 wt % CaSiO_3_ (**c**,**d**) for three days (**a**,**c**) and five days (**b**,**d**).

The cells well spread with abundant extracellular matrix on the scaffolds with 20 wt % CaSiO_3_ after being cultured for three days (Figure 10), which was similar to those cultured for five days on the scaffolds without CaSiO_3_. After five days, a high-density cell fusion formed multiple layers with some folds on the scaffolds. The results suggested that the scaffolds with CaSiO_3_ possessed the ability to support cell adherence and proliferation. It was believed that the degradation of CaSiO_3_ could form a relative rough surface which was favorable for cell adhesion. On the other hand, the silicon, calcium and magnesium ionic products dissolved from the scaffold at certain concentration range were also shown to have a positive stimulatory effect on promoting cell proliferation and inducing cell differentiation [9].

**Figure 11:**
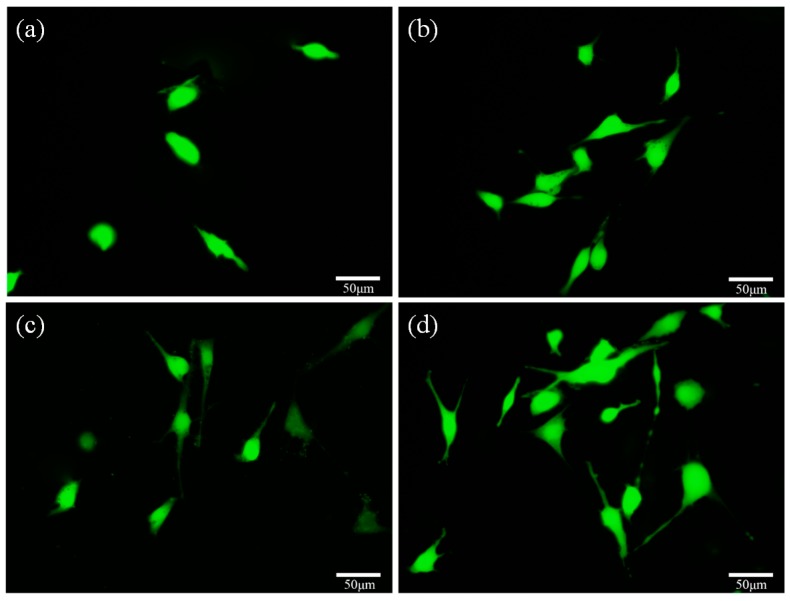
Fluorescence microscopies of MG63 cells cultured on scaffolds without CaSiO_3_ (**a**,**b**) and scaffolds with 20 wt % CaSiO_3_ (**c**,**d**) for three days (**a**,**c**) and five days (**b**,**d**).

Fluorescence microscopies of cells cultured on the scaffolds revealed that the live cells on the scaffolds appear as green spots (Figure 11). It was clear to see that the viable cells attached well and spread with the extended filopodia on the scaffolds with 20 wt % CaSiO_3_ after being cultured for three days (Figure 11c), which was similar to those cultured for five days on the scaffolds without CaSiO_3_. With the culture time prolonged to five days, the number of cells increased, the cell filopodia further extended, and some cell fusion appeared (Figure 11d). The fluorescence results were accorded with the development as shown in Figure 10, which further demonstrated that the scaffolds with CaSiO_3_ had a stimulatory effect on promoting cell attachment and proliferation.

## 3. Materials and Methods

### 3.1. Scaffold Fabrication

Mg_2_SiO_4_, which was provided by Alfa Aesar China Co., Ltd. (Tianjin, China), was medical grade material with an average particle size of about 5 μm. CaSiO_3_, which was provided by Kunshan Huaqiao New Materials Co., Ltd. (Kunshan, China), had a diameter of 0.2–2 μm. The Mg_2_SiO_4_ and CaSiO_3_ powders were mixed in different proportions using the ultrasonic method and the ball mill method. In detail, the Mg_2_SiO_4_ and CaSiO_3_ powders were first dispersed in ethanol for 30 min of ultrasonication, and then followed by 30 min of ball mill grinding at room temperature using a variable frequency planet-type grinding mill. Three different-diameter ZrO_2_ balls (3, 5 and 10 mm) were mixed as the milling media to enhance the homogeneity. After milling, the obtained powders were dried in a draught drying cabinet at 70 °C for 12 h.

The prepared mixed powders were used as sintering raw materials for fabricating the porous scaffolds via SLS. The SLS system contained a laser with an optical focusing system, sintering platform, three-dimensional motion platform and the control system [29]. In the SLS process, the mixed powders were selectively sintered layer by layer to form the interconnected porous scaffold. The relevant process parameters were kept constant as follows: laser power of 8.5 W; spot diameter of 1 mm; scanning rate of 100 mm/min; scanning line interval of 3.5 mm; and average layer thickness of 0.1 mm.

### 3.2. Scaffold Characterization

The microtopography of the scaffolds was studied by scanning electron microscopy (SEM) (TESCAN MIRA3 LMU, Co., Brno, Czech Republic) completed with energy dispersive spectroscopy (EDS). Before SEM observation, the scaffolds were treated by desiccation and spray platinum (JFC-1600, JEOL Co., Tokyo, Japan). The phase composition of the scaffolds were determined by X-ray diffraction (XRD) (Rigaku Co., Tokyo, Japan) using a Cu-Kα source (λ = 1.5418 Å) in a range of 10° to 80° with 8°/min scanning rate.

The compression strength of the scaffolds was measured using a microcomputer-controlled electron universal testing machine (WD-D1, Shanghai Zhuoji Instruments Co., Ltd., Shanghai, China). The compression load was applied on scaffolds at a speed of 0.5 mm/min until the scaffolds were completely crushed. The compression strength was calculated by dividing the peak load by the cross-sectional area of the scaffolds. The fracture toughness of the scaffolds was measured using a digital microhardness tester (Shanghai Taming Optical Instrument Co., Shanghai, China). A load of 4.98 N was applied on polished surface for 10 s by a pyramid-shaped diamond indenter to induce indentations and cracks. Then the load and the crack length were used to calculate the fracture toughness (*K_IC_*) based on the following Equation (3) [30]:

(3) $$K_{IC}=0.0824P\times C^{-3/2}$$

where *P* is the load applied by the indenter and *C* is the length of diagonal crack.

### 3.3. Degradation Behavior

The degradability of the scaffolds was evaluated based on their weight loss in phosphate buffer saline solution (PBS; pH = 7.4). The scaffolds were soaked in the solution for different periods (7, 14, 21, and 28 days) in an incubator at 37 °C, and the solution was renewed every 24 h. After the predetermined soaking time, the scaffolds were taken out from the solution, carefully rinsed in ethanol and then desiccated at 70 °C for 10 h. The weight of the scaffolds was accurately measured before and after soaking.

### 3.4. Bioactivity

Bioactivity of the scaffolds was assessed by detecting the formation of bone-like apatite after soaking in simulated body fluid (SBF). The SBF solution had a similar composition and concentrations to that of human blood plasma (Table 1) [31]. The scaffolds were soaked in SBF for 14 days in an incubator with a constant temperature of 37 °C, and the SBF solutions were replaced every other day. After soaking, the scaffolds were carefully rinsed in ethanol and then desiccated at room temperature for further characterization. The morphology and chemical group of bone-like apatite formed on the scaffolds were characterized by SEM and Fourier transform infrared spectroscopy (FTIR) (Thermo Electron Scientific Instruments, Madison, WI, USA), respectively.

### 3.5. Cell Culture

Cytocompatibility of the scaffolds was assessed by MG63 osteoblast-like cell (Cellular Biology Institute, Shanghai, China) culture studies in 12-well culture plate. Prior to the cell culture, the scaffolds were immersed in ethyl alcohol solution for 2 h of sterilization, then exposed to ultraviolet light for 15 min and washed with PBS. After that, they were seeded with MG63 cell (20,000 cells/well) and cultured in Dulbecco’s Modified Eagle’s Medium (DMEM) with 10% (*v*/*v*) fetal bovine serum at 37 °C in a 5% CO_2_ humid incubator with the culture media refreshed every three days. After different culture times (three days, five days), the scaffolds were removed from the culture plate and rinsed with PBS to remove the unattached cells. Then, the adherent cells on the scaffolds were fixed with 4% glutaraldehyde (for 30 min) and successively dehydrated in ethanol (70.0%, 80.0%, 90.0% and 100.0%). Afterwards, the scaffolds were dried and sputter-coated with platinum. Finally, the cell-scaffold interactions and cell behavior were visualized using SEM.

Moreover, the cell-scaffolds interaction was also investigated using fluorescence technique. After different culture times, scaffolds were taken out from the culture plate and cleaned with PBS, then settled with buffered ice-cold paraformaldehyde and permeabilized with 0.1% Tween 20. Afterwards, the cells were rinsed again with PBS and preincubated by 1% gelatin in PBS. Subsequently, the cells were incubated for 30 min in the compound of 4 μM EthD-1 and 2 μM calcein AM in PBS. Finally, Fluorescence microscopies of the cells were visualized using confocal microscope.

### 3.6. Statistical Analysis

All the data were statistically analyzed using one-way analysis of variance (ANOVA) and shown as mean ± standard deviation (*N* = 5) where appropriate. The level of statistically significance was set at *p*-value less than 0.05.

## 4. Conclusions

In this study, a MgO-CaO-SiO_2_ system was developed with Mg_2_SiO_4_ and CaSiO_3_ to fabricate scaffolds. The optimal mechanical properties were obtained when incorporated 20 wt % CaSiO_3_. The reason for this was that the liquid phase of CaSiO_3_ enhanced the densification. The degradability and bioactivity of the scaffolds improved with increasing amounts of CaSiO_3_. Furthermore, cell culture experiments indicated that the scaffolds with CaSiO_3_ supported the adhesion and proliferation of MG63 cells. Therefore, the scaffolds with 20 wt % CaSiO_3_ might be promising candidates as bone implants.

## Figures and Tables

**Table 1:** Ion concentration in SBF and human blood plasma.

Ion Type	Ion Concentration/(mmol/L)						
	Na^+^	K^+^	Mg^2+^	Ca^2+^	Cl^−^	${\text{HCO}}_{3}^{-}$	${\text{HPO}}_{4}^{2-}$
Blood Plasma	142.0	5.0	1.5	2.5	103.0	17.0	1.0
SBF	142.0	5.0	1.5	2.5	148.8	4.2	1.0
